# Electrically pumped quantum-dot lasers grown on 300 mm patterned Si photonic wafers

**DOI:** 10.1038/s41377-022-00982-7

**Published:** 2022-10-14

**Authors:** Chen Shang, Kaiyin Feng, Eamonn T. Hughes, Andrew Clark, Mukul Debnath, Rosalyn Koscica, Gerald Leake, Joshua Herman, David Harame, Peter Ludewig, Yating Wan, John E. Bowers

**Affiliations:** 1 Materials Department, University of California Santa Barbara, Santa Barbara, CA 93106 USA; 2 Department of Electrical and Computer Engineering, University of California Santa Barbara, Santa Barbara, CA 93106 USA; 3 IQE, Inc., Greensboro, NC 27409 USA; 4 RF SUNY Polytechnic Institute, Albany, NY 12203 USA; 5 NAsPIII/V GmbH, Marburg, Germany

**Keywords:** Semiconductor lasers, Silicon photonics, Diode lasers

## Abstract

Monolithic integration of quantum dot (QD) gain materials onto Si photonic platforms via direct epitaxial growth is a promising solution for on-chip light sources. Recent developments have demonstrated superior device reliability in blanket hetero-epitaxy of III–V devices on Si at elevated temperatures. Yet, thick, defect management epi designs prevent vertical light coupling from the gain region to the Si-on-Insulator waveguides. Here, we demonstrate the first electrically pumped QD lasers grown by molecular beam epitaxy on a 300 mm patterned (001) Si wafer with a butt-coupled configuration. Unique growth and fabrication challenges imposed by the template architecture have been resolved, contributing to continuous wave lasing to 60 °C and a maximum double-side output power of 126.6 mW at 20 °C with a double-side wall-plug efficiency of 8.6%. The potential for robust on-chip laser operation and efficient low-loss light coupling to Si photonic circuits makes this heteroepitaxial integration platform on Si promising for scalable and low-cost mass production.

## Introduction

Internet data traffic has seen a compound annual growth rate of 27% in the last few years and exceeded one zettabyte in 2017. Leveraging established CMOS processing, Si photonics promises to fulfill this soaring demand for high volume applications such as more efficient, higher capacity, and lower cost interconnects with numerous successes in high-performance passive components on 300 mm Si^1–3^. However, due to the indirect bandgap nature of Si and Ge, integrating III–V lasers with Si photonics is a vital step toward widespread adoption. Mass production has been achieved using wafer bonding of III–V gain regions to Si-on-Insulator (SOI) wafers where light is evanescently coupled to the Si waveguide underneath^4–6^. Alternatively, direct growth of III–V gain material onto Si substrates may prove to be a more economically favorable solution, which not only eliminates the need for III–V wafers and the complex bonding process, but also provides the additional benefits of compact packaging and better heat sinking. The main challenge is the inevitable crystalline defects that form when merging the III–V and Si crystals via epitaxial growth. To date, tremendous advancements have been made in blanket hetero-epitaxy of III–V compound semiconductors on Si substrates. Anti-phase domains (APDs) from growing polar III–V onto non-polar single step (001) Si have been solved with sophisticated surface preparation, either by utilizing etch high index Si surfaces^7–12^ or by inducing double step edges and carefully control of the initial III–V nucleation process^13–16^. Threading dislocations that nucleate as the III–V film relaxes due to the lattice mismatch are thought to be the most detrimental to devise performance as they penetrate through all layers. A considerable amount of effort has been demoted to reduce the threading dislocation density (TDD)^17–19^. Recently, asymmetric step-graded filter was developed such that the TDD has been reduced to as low as 1 × 10^6^ cm^−2^ within a 2.55 µm GaAs virtual substrate grown on (001) Si^20^. However, film cracking and wafer bowing are more prominent as TDD is further reduced^21^. The coefficient of thermal expansion has recently been discovered to be responsible for the formation of misfit dislocations (MDs) near the active region, as the film stress state changes from compression to tension during the post-growth cooling process. In lasers with low TDD, the MDs are more detrimental to the device reliability than TDs considering their larger interaction area with the active region^22^. To combat with the MDs, thin strained quantum wells (QWs) were inserted as trapping layers above and below the active region to block the MDs away from the active region^23^. This research field was further boosted by using a quantum dot (QD) active region in place of the traditional QWs. High-performance electrically pumped epitaxial devices were then realized on offcut Si substrate^24,25^. Due to the atom-like density of states, switching to QDs as active not only provides a lower entry to Si photonics thanks to the greatly reduced sensitivity to non-radiative defects^26,27^, but also offers numerous unique properties that are beneficial to photonic integrated circuits. Great strides have been made in individual QD devices grown on Si, showing high temperature stability^28^, low threshold operation^26,29^, and low reflection sensitivity^30^, etc. As a result of the parallel effort in these defect management advances on (001) Si and innovations in the active region, the extrapolated lifetime of epitaxially grown QD lasers by molecular beam epitaxy (MBE) on CMOS compatible blanket Si device show minimum degradation after more than 4000 h of constant current stress at 80 °C, with an extrapolated lifetime exceeding 200,000 h^31^.

However, the thick buffer layers required to reduce the TDD prevent evanescent coupling to the underlying Si waveguides and have hindered the integration of epitaxial QD lasers with conventional Si photonics. One solution is to grow the III–V gain material in narrow pockets and butt-coupled to SiN or SOI waveguides embedded in the surrounding matrix material. Such an approach offers the densest integration of the on-chip gain elements with an advanced Si photonic platform. Dense integration, together with the flexible positioning, of amplifiers and lasers is critical for datacom and optical phased array-based LiDAR. It has also been inferred that growing directly in narrow pockets would provide another path for performance enhancement due to the unique stress profile provided by the pocket geometry^21,32^.

Passive waveguide coupling^33^ or optically pumped nano-ridge lasers on patterned 300 mm Si^34^ have previously been demonstrated. The vertical alignment of the QD active region to the waveguides can be precisely controlled during growth. Such in-pocket configuration also mitigates the issues with the residual tension, possibly yielding lasers with higher reliability compared to those grown on blanket Si^21,32^. However, transferring the epi stack, especially the QD active region, from the blanket Si substrate into the pockets on the patterned Si template is a non-trivial task. In this work, we have identified and solved the additional growth complications induced by the template architecture and have achieved high-quality QD nucleation in the pockets. As a proof of concept for this integrated photonic platform with a CMOS process on a 300-mm Si wafer, we demonstrate, to the best of our knowledge, the first electrically pumped in-pocket Fabry–Perot QD lasers grown by MBE emitting around 1300 nm with cleaved facets, sustaining lasing characteristics up to 60 °C with a wall-plug efficiency of 8.6%.

## Results

**Fig. 1:**
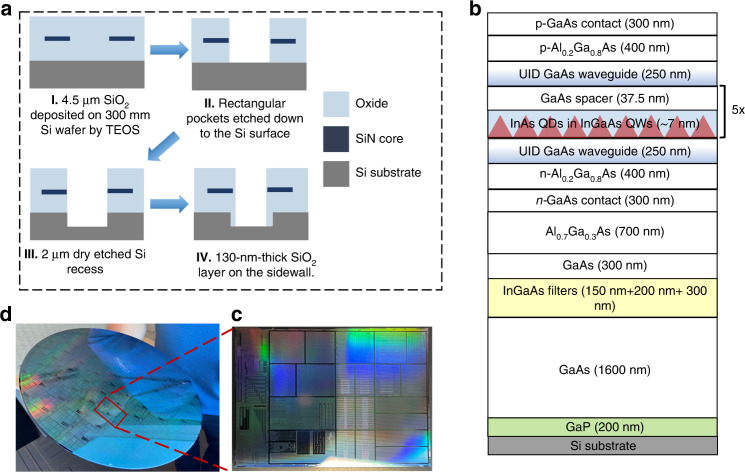
Template preparation and proposed laser stack. **a** Schematic diagram of the pattern formation process before III–V deposition. **b** Simplified III–V laser stack. **c** The diced coupon from the as-patterned 300 mm Si wafer for growth condition investigation. **d** As-patterned 300 mm Si wafer.

Figure 1a depicts the fabrication process of the 300 mm Si photonic template with tetraethoxysilane (TEOS) oxide pattern prior to depositing the III–V QD laser epi structure, as shown in Fig. 1b. The details could be found in Materials and methods.

**Fig. 2:**
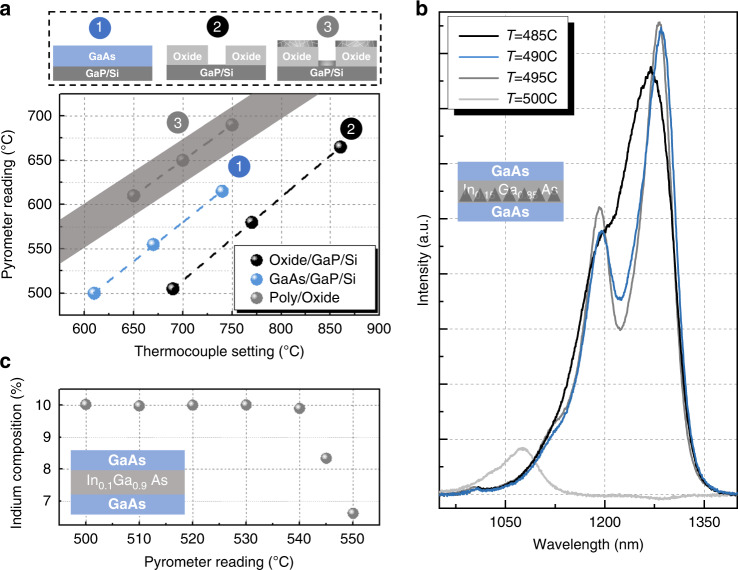
Identification of the temperature offset window. **a** Pyrometer reading discrepancy between different surface conditions. The shaded area indicates the range of readings obtained depending on the polycrystal thickness, with the lower and the upper boundaries indicating the reading measured at the beginning and the end of MBE deposition, respectively. The points in profile 3 represent pyrometer measurements obtained just before InAs QD deposition. **b** PL growth temperature series on native GaAs substrate. **c** Indium composition as a function of growth temperature. The insets of (b) and (c) illustrate the test sample structures.

**Fig. 3:**
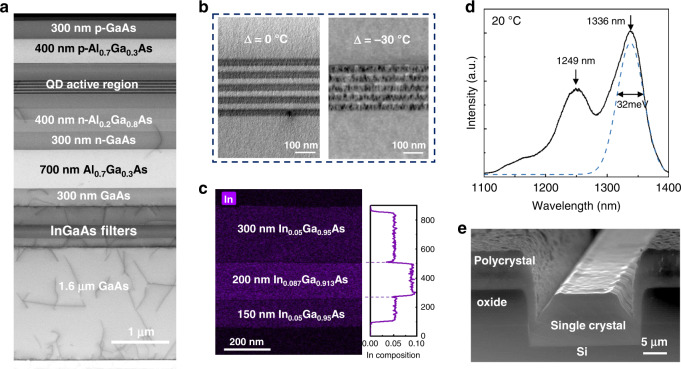
Characterization of the as-grown in-pocket material. **a** Cross-section TEM of the full in-pocket III–V stack above the 200 nm GaP. **b** Cross-sectional STEM of the active region with different temperature adjustments. Clear QD contrast observed after 30 °C decrease (right). **c** EDS map of the dislocation filter layers labeled with the designed compositions (left) and the extracted actual composition (right). **d** Room-temperature PL spectrum from the in-pocket laser material, the dashes line is the Gaussian fit of the ground state line shape. **e** Cross-section SEM of the as-grown in-pocket material.

Achieving the goal of an electrically pumped QD laser requires a precise understanding of growth condition drifts due to the patterned oxide surrounding the pockets and the resulting III–V epitaxy quality. This work was undertaken using coupons (Fig. 1c) diced from an equivalent 300 mm Si template (Fig. 1d) that were transferred to a Veeco Gen-II 3-inch solid source MBE system at UCSB for depositing the same QD laser structure described above. Since the majority of the template area is covered by the oxide mask, using reflective high-energy electron diffraction as the in situ monitor for the surface quality and QD nucleation is not feasible, and the pyrometer readings are less trustworthy. As shown in Fig. 2a, we observed that, for the same sample geometry, the sample with the oxide mask gives a lower pyrometer reading compared to that measured on blanket GaAs/GaP/Si at the same heater power due to the lower emissivity of the oxide. After the oxide mask is covered with polycrystalline III–V from the non-selective growth, the pyrometer reading is higher than that measured on blanket GaAs/GaP/Si. The thicker the polycrystalline layer, the higher the pyrometer reading, suggesting the pyrometer reading depends on the thermal mass of the absorptive polycrystal III–V. Though selective area MBE growth can be realized with no polycrystal deposition on the oxide mask, the required high growth temperatures and low growth rate would degrade the QD nucleation^35^. Thus, trusting pyrometer profiles calibrated from either the Si template or the polycrystalline-covered surface before InAs QD deposition would result in either an under- or over-estimate of the actual growth temperature, respectively. Since the optimal temperature window for QD nucleation is small (±2.5 °C), this template architecture introduces large temperature uncertainty, making it challenging to achieve high-quality QD nucleation. Therefore, sample heater power calibrated on a blanket GaAs/GaP/Si template was used for the pocket growth instead of the pyrometer, and blanket-substrate-level III–V film quality was achieved with accurate layer thicknesses, shown later in Fig. 3a. The TDD estimated from cross-sectional scanning transmission electron microscopy (STEM) is 1.5 × 10^7^ cm^−2^ above the InGaAs dislocation filter layers without utilizing the extensive thermal cyclic annealing after depositing the initial 1.6 μm GaAs. The filtering efficiency is then estimated to be 98% given that the surface TDD after the initial 1.6 μm GaAs is 6 × 10^8^ cm^−2^, measured with plan-view TEM (not shown).

However, no QD contrast was observed in the cross-section TEM, shown in Fig. 3b (left), and no photoluminescence (PL) signal was obtained from this early structure. Thus, it appears that the same heater power that achieves a surface temperature of 495 °C for QD nucleation on blanket GaAs/GaP/Si results in a higher temperature in the pockets, potentially evaporating the InAs QD material. We attribute this to more radiation being absorbed by the highly defective polycrystalline III–V from the ambient and less heat being radiated from the mask-covered area due to the lower oxide thermal conductivity. To obtain an estimate of the overheating window, separate experiments were conducted on native GaAs substrates. Test structures mimicking the laser active region with one layer of QDs were grown at various pyrometer temperatures. As shown in Fig. 2b, the PL spectrum obtained suggests that an overheating of merely 5 °C above the optimal nucleation temperature would result in severe PL signal degradation due to the evaporated dots. To obtain the upper bound of the template-induced overheating, test structures on GaAs substrates with 50 nm In_0.1_Ga_0.9_As, capped with 10 nm GaAs were grown at different pyrometer temperatures. A loss of indium was observed at a growth temperature above 540–545 °C, as shown in Fig. 2c.

A STEM EDS map of the in-pocket InGaAs filter layers was then obtained, showing that the InGaAs filters preserve the designed indium composition, shown in Fig. 3c. This suggests that the overheating from the template architecture is no more than 45 °C. Lowering the heater power was then carried out in 5 °C intervals from the calibrated pyrometer profile obtained on the blanket GaAs/GaP/Si wafer. High-quality QD nucleation was finally achieved after an approximately 30 °C decrease, shown in Fig. 3b (right). Room-temperature PL signal, shown in Fig. 3d, was then obtained from the in-pocket material with a ground state peak near 1300 nm, a full width at half maximum of 32 meV, and a ground-to-excited-state peak separation of 70 meV, comparable to typical values for blanket Si substrates. The above growth conditions form the basis for the process transfer to a 300 mm MBE reactor at IQE, Inc. with similar process trends observed on the larger production platform. The laser results reported here were grown at IQE on 300 mm wafers.

**Fig. 4:**
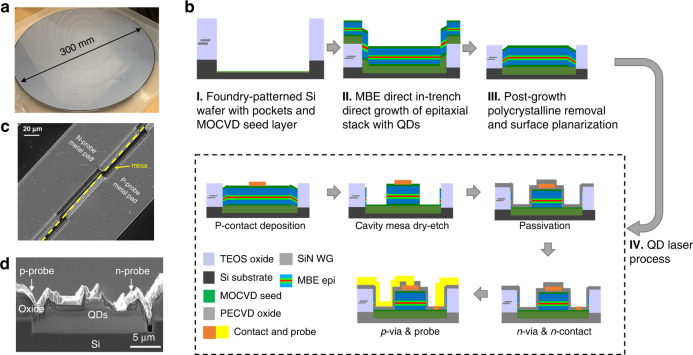
Fabrication process of the in-pocket laser. **a** As-grown 300 mm wafer from IQE, covered with polycrystal III–V. **b** Simplified fabrication flow for laser fabrication, not drawn to scale. **c** Top-down view of an as-fabricated device. **d** Cross-section SEM of the as-cleaved in-pocket laser.

The as-grown 300 mm wafer, shown in Fig. 4a, was diced into rectangular coupons for processing at the UCSB Nanofab facility. The surface morphology presents unique challenges for device fabrication due to the non-selectively deposited polycrystalline III–V on the oxide surface and the trench sidewalls, as well as heavy faceting near the trench edge, previously shown in Fig. 3c. Process development steps were undertaken to transfer processes for blanket-wafer ridge waveguide lasers to fabrication of the in-pocket gain section. Figure 4b shows schematics of the fabrication process flow. We developed a wet etch process to selectively remove the polycrystalline III–V on the oxide surface. The removal of surface polycrystal enables easier handling with a more planarized sample surface and better focus calibration in the photolithography steps. Planarized sample surfaces also serve for better metal liftoff. The alignments were carried out using markers pre-patterned in the SiN waveguide layers on the Si handle wafer. Ridge waveguides were defined with a Cl_2_-based one-step inductively coupled plasma etch that exposes the n-doped GaAs contact layer. Thick PECVD SiO_2_ was used for passivation of waveguide sidewalls as well as pocket edges. We deposited Pd/Ti/Pd/Au for p-contact at the ridge-top, and Pd/Ge/Au for n-contact on top of the bottom n-doped layer and cladding layer, respectively. After that, we used dry-etching for via-opening and performed probe-metal deposition to form probing pads at the wafer surface that are routed toward contacts inside the pockets.

Figure 4c shows the completed chip with probe metal along the gain-element pockets. To investigate the QD laser quality as a proof of concept, Fabry–Perot cavities were formed with as-cleaved facets along the <110> directions after Si substrate thinning. No further facet coating was applied at this point. Figure 4d shows a cross-section SEM of the image of a device in a pocket of width around 20 µm. The device has a ridge width of 3.5 µm, and the bright probe-metal layer shows the routing across the pocket-edge step of over 4 µm, demonstrating the feasibility of testing active elements inside pockets from the wafer surface despite the challenges from the high aspect ratio of the device geometry.

**Fig. 5:**
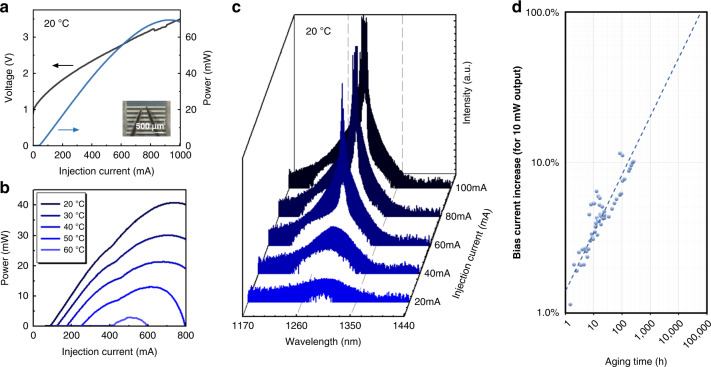
Laser measurement results. **a** Room-temperature LIV of the device with the highest output power. Inset shows the probe needles on a cleaved laser bar. **b** Temperature-dependent LIV shows lasing up to 60 °C. **c** Lasing spectrum as a function of injection current at room temperature. **d** Aging results for the in-pocket laser at 35 °C.

Electrically pumped lasing was achieved under CW conditions with a maximum double-side output power of 126.6 mW at a stage temperature of 20 °C, a threshold current of 47.5 mA, and a series resistance of 3.5 ohms. The performance is comparable to devices grown on blanket Si substrate with similar TDD^36^. The highest double-side wall-plug efficiency of 8.6% is achieved at an injection current of 214 mA. Figure 5a shows the light–current–voltage (*L–I–V*) of this device. Devices with comparable performance were obtained from different pocket widths on the same cleaved device bar, such as 20 and 30 µm, which indicates relatively high crystal quality and low geometry-dependent growth rate variation owning to the ballistic nature of MBE growth. Lasers from other process runs and from QDs grown on smaller wafers at UCSB showed similar results. Temperature-dependent measurements of the lasers were also carried out, with *L–I–V* curves of a 2 mm long device shown in Fig. 5b. CW lasing was observed up to 60 °C (pulsed lasing up to 70 °C). Compared to ridge waveguide QD lasers grown on blanket Si wafer, the heat dissipation is potentially limited by the thick oxide layer on the sample surface near the device ridge along the pockets. However, we expect the high-temperature device performance to be improved from further optimization in various aspects, such as template quality, growth condition, and device design.

To study the lasing wavelength, we aligned a lensed fiber at the cleaved facet to collect the spectra with an optical spectrum analyzer (Yokogawa, AQ6370C). The spectra of a 2 mm long device with 4.5 µm ridge width at various injection currents at a stage temperature of 20 °C are shown in Fig. 5c. Below threshold, the amplified spontaneous emission spectrum shows the peak at around 1300 nm, where lasing was achieved beyond threshold with a peak at 1300 nm. A slight red shift in the lasing wavelength has been observed as the injection current increases due to junction heating. The lasing wavelength agrees well with the in-pocket PL obtained after MBE growth prior to fabrication. A representative device was then aged continuously at two times the initial threshold current at 35 °C and the L–I curves were obtained at each testing interval. The change in the bias required for 10 mW output power for the first 350 h is shown in Fig. 5d. The aging performance of the in-pocket laser is comparable to those grown on blanket Si substrate with similar TDD^36^, reaffirming blanket-substrate-level crystal and QD quality. In-depth analysis of the degradation mode, together with designing stacks for better defect management in the pockets, is beyond the scope of this work.

The in-pocket gain-element fabrication process can be adapted and transferred to different epitaxial layer stack designs in the AlGaAs/GaAs material system to be used in this on-chip butt-coupled heteroepitaxial integration platform. With additional lithography and etching steps and/or alternative layout designs, a variety of active components such as semiconductor optical amplifiers, mode-locked lasers, or distributed-feedback lasers can also be fabricated from the same chip. This fabrication process can be used in integrating complex photonic circuits on 300 mm diameter SOI wafers.

## Discussion

**Fig. 6:**
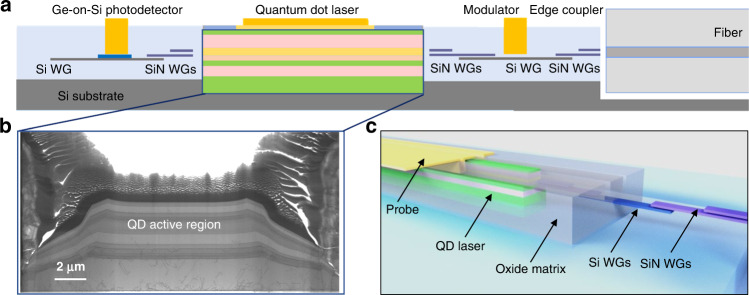
Heteroepitaxial laser integration on Si photonic platform. **a** Schematic diagram of the heteroepitaxial laser integration on Si platform. **b** Cross-section TEM of full laser epi in the pockets grown on 300 mm Si from IQE. **c** Schematic of intended device configuration.

In summary, we present here the first electrically pumped QD lasers epitaxially grown in pockets of patterned 300 mm Si photonic wafers. A maximum double-side output power of 126.6 mW at 20 °C and CW lasing up to 60 °C were observed. Identifying the MBE growth challenges introduced by the template architecture paves the way for high-quality QD nucleation, with near-blanket-level crystalline quality. It is worth mentioning that the growth conditions, especially the QD nucleation temperature offset, are expected to be dependent on the oxide pattern structure, pattern fill factor, and polycrystal thickness. Thus, each specific combination of template and epi design would potentially require a specific set of growth conditions and so standardized pocket sizes and pattern designs are desirable. Fortunately, the proposed method for locating the optimal growth window is applicable to other combinations of templates and epi designs. The key challenge would then be identifying the most temperature-sensitive crystalline equality of the specific epi design, including, but not limited to composition and morphology. Selective polycrystal removal neutralizes the potential issue with lithography and metal continuity from the thick non-selective III–V deposition on the oxide mask, resulting in the highly conformal metal routing around the in-pocket laser strip. The in-pocket QD lasers in this work are a promising approach to integrate on-chip gains on existing Si photonic platforms, as schematically illustrated in Fig. 6. The eventual realization of large-scale, low-cost, and high-density monolithically integrated lasers and amplifiers on Si via direct epitaxial growth would enable the next generation of photonics integrated circuit platforms.

## Materials and methods

The initial 4.5 µm SiO_2_ was deposited by low-frequency plasma chemical vapor deposition using TEOS on CMOS compactible 300 mm Si wafers. Rectangular pockets were then dry etched down to the Si surface with a pattern fill factor of 95%, followed by a Si recess to make room for the defect-managing buffer layers with InGaAs dislocation filters. To protect the recessed Si sidewall and avoid lateral growth of III–V in the pocket, a thin SiO_2_ layer was then deposited on the sidewall. The in-pocket Si surface was then etched back with HCl and selectively refilled to the previous level after a post-etching high-temperature heat treatment in the reactor. A thin 200 nm APD-free GaP layer followed by 500 nm of GaAs was then selectively deposited in a 300 mm MOCVD reactor at NAsP_III/V_ GmbH as a seed layer^37^. The intended buffer structure for the QD laser consisted of 1.1-μm thick GaAs, followed by the InGaAs asymmetric graded dislocation filter layers grown at 500 °C. The filter structure consists of a 200 nm In_0.1_Ga_0.9_As sandwiched between two In_0.05_Ga_0.95_As layers (150 nm below and 300 nm above). The buffer structure was then completed with an additional 300 nm of GaAs^20^. The active region consisted of five layers of InAs QDs, nominally grown at 495 °C on blanket substrates, embedded in 7 nm In_0.15_Ga_0.85_As QWs with 40 nm GaAs barriers. Surrounding the active region were the AlGaAs cladding regions and the *n*- and *p*-GaAs contact layers.

## Data Availability

Data may be obtained from the authors upon reasonable request.
